# Angiogenic and Vasculogenic Factors in the Vitreous from Patients with Proliferative Diabetic Retinopathy

**DOI:** 10.1155/2013/539658

**Published:** 2013-03-10

**Authors:** Ahmed M. Abu El-Asrar, Mohd Imtiaz Nawaz, Dustan Kangave, Mohammed Mairaj Siddiquei, Karel Geboes

**Affiliations:** ^1^Department of Ophthalmology, College of Medicine, King Saud University, Riyadh, Saudi Arabia; ^2^Department of Ophthalmology, King Abdulaziz University Hospital, Old Airport Road, P.O. Box 245, Riyadh 11411, Saudi Arabia; ^3^Laboratory of Histochemistry and Cytochemistry, University of Leuven, Belgium

## Abstract

This study was conducted to determine levels of angiogenic and endothelial progenitor cell mobilizing (vasculogenic) factors in vitreous fluid from proliferative diabetic retinopathy (PDR) patients and correlate their levels with clinical disease activity. Vascular endothelial growth factor (VEGF), soluble vascular endothelial growth factor receptor-2 (sVEGFR-2), stem cell factor (SCF), soluble c-kit (s-kit), endothelial nitric oxide synthase (eNOS), and prostaglandin E_2_ (PGE_2_) levels were measured by ELISA in vitreous samples from 34 PDR and 15 nondiabetic patients. eNOS was not detected. VEGF, sVEGFR-2, SCF, and s-kit levels were significantly higher in PDR with active neovascularization compared with quiescent PDR and nondiabetic patients (*P* < 0.001; 0.007; 0.001; <0.001, resp.). In contrast, PGE_2_ levels were significantly higher in nondiabetic patients compared with PDR patients (*P* < 0.001). There were significant correlations between levels of sVEGFR-2 versus SCF (*r* = 0.950, *P* < 0.001), sVEGFR-2 versus s-kit (*r* = 0.941, *P* < 0.001), and SCF versus s-kit (*r* = 0.970, *P* < 0.001). Our findings suggest that upregulation of VEGF, sVEGFR-2, SCF, and s-kit supports the contributions of angiogenesis and vasculogenesis in pathogenesis of PDR.

## 1. Introduction

 Angiogenesis, the process by which new vascular networks develop from preexisting vessels, is a hallmark feature of proliferative diabetic retinopathy (PDR). In addition, increasing evidence suggests that vasculogenesis, the de novo formation of blood vessels from circulating bone marrow-derived endothelial progenitor cells (EPCs), can contribute to neovascularization. Recent studies have shown that circulating bone marrow-derived EPCs home to the ischemic region, differentiate into mature endothelial cells in situ, and can contribute to the process of neovascularization [1, 2]. In previous studies, we demonstrated that bone marrow-derived CD133^+^ EPCs and c-kit^+^ cells contribute to the new vessel formation in PDR fibrovascular epiretinal membranes [3, 4].

 Angiogenesis and vasculogenesis are dependent on several cytokines/chemokines and their associated tyrosine kinase receptors. A key player of both these processes is vascular endothelial growth factor (VEGF), also called vascular permeability factor [5, 6]. VEGF binds with high affinity and activates two tyrosine kinase receptors, VEGFR-1 (Flt-1) and VEGFR-2 (KDR in humans/Flk-1 in mice). These receptors regulate physiological as well as pathological angiogenesis. From the postnatal to adult stage, VEGFR-2 is expressed mostly on vascular endothelial cells [7]. VEGFR-2 is also expressed by bone marrow-derived circulating EPCs. EPCs are characterized by the expression of markers like CD133, CD34, and VEGFR-2 [1, 2]. VEGFR-2 has strong tyrosine kinase activity and is the major positive signal transducer for pathological angiogenesis including cancer and diabetic retinopathy as well as microvascular permeability [7]. Activation of VEGFR-2 stimulates endothelial cell proliferation, migration, and survival, as well as angiogenesis and microvascular permeability [7]. VEGFR-2 has a truncated soluble form (sVEGFR-2) that can be detected in mouse and human plasma. However, it is unknown whether the sVEGFR-2 is a product of ectodomain shedding from cell-surface VEGFR-2 or a product of alternative mRNA splice variation [8, 9].

 Stem cell factor (SCF) or kit ligand is a peptide growth factor that exists as a membrane-bound protein but may be cleaved by proteases such as matrix metalloproteinase-9 (MMP-9), to produce the soluble form [10–12]. SCF is important for the survival, proliferation, and differentiation of hematopoietic stem cells. The receptor for SCF, the proto-oncogene c-kit is a tyrosine kinase that is expressed by bone marrow-derived endothelial stem/progenitor cells that can give rise to endothelial cells [13, 14]. SCF ligand binding leads to phosphorylation and activation of the c-kit receptor and its downstream signaling proteins which have been implicated in cell proliferation, cell adhesion, cell survival, chemotaxis, and mobilization of EPCs required for neovascularization [11, 12, 15]. SCF/c-kit signaling has been implicated in the regulation of angiogenesis [10, 13, 15–18]. A soluble form of c-kit (s-kit), consisting of only the extracellular ligand-binding domain, that can be generated by proteolytic cleavage from the surface of hematopoietic cells, mast cells, and endothelial cells or by alternative splicing has been identified [19].

Several studies reported that endothelial nitric oxide synthase (eNOS) is crucial for the recruitment of EPCs in the circulation from the bone marrow and for firm c-kit^+^ cell adhesion to the vascular endothelium. eNOS is also required for neovascularization in ischemic tissue [20–23]. Recently, it was reported that prostaglandin E_2_ (PGE_2_), one of the major products of cyclooxygenase, plays an essential role in EPCs homeostasis [24]. In addition, PGE_2_ directly stimulates angiogenesis, apart from VEGF signaling, and further induces VEGF expression in endothelial cells [25].

 We hypothesized that the vitreous levels of these biomarkers directly reflects angiogenesis and vasculogenesis in PDR. To elucidate the role of angiogenic and EPC mobilizing factors in PDR progression, we measured the levels of VEGF, sVEGFR-2, SCF, s-kit, eNOS, and PGE_2_ in the vitreous fluid from patients with PDR and patients without diabetes and correlated their levels with clinical disease activity. 

## 2. Materials and Methods

### 2.1. Vitreous Samples Collection and Preparation

Undiluted vitreous fluid samples (0.3–0.6 mL) were obtained from individual eyes of from 34 patients with PDR during pars plana vitrectomy. The indications for vitrectomy were traction retinal detachment and/or nonclearing vitreous hemorrhage. The severity of retinal neovascular activity was graded clinically at the time of vitrectomy using previously published criteria [26]. Neovascularization was considered active if there were visible perfused new vessels on the retina or optic disc present within tractional epiretinal membranes. Neovascularization was considered inactive (involuted) if only nonvascularized; white fibrotic epiretinal membranes were present. Active PDR was present in 21 patients, and inactive PDR was present in 13 patients. Traction retinal detachment was present in 23 patients and vitreous hemorrhage in 16 patients. Vitreous hemorrhage was present in 12 patients with active PDR and in 4 patients with inactive PDR. The diabetic patients were 24 males and 10 females, whose ages ranged from 27 to 75 years with a mean of 53.3 ± 11.7 years. The duration of diabetes ranged from 7 to 32 years with a mean of 16.4 ± 5.6 years. Twenty-two patients had insulin-dependent diabetes mellitus, and 12 patients had noninsulin-dependent diabetes mellitus. At presentation, the fasting blood glucose was uncontrolled in 22 patients and controlled in 12 patients. Twenty-four patients were receiving treatment for hypertension, 3 patients had diabetic nephropathy, and 3 patients had cardiovascular disease. The control group consisted of 15 patients who had undergone vitrectomy for the treatment of rhegmatogenous retinal detachment (RD) with no proliferative vitreoretinopathy. Controls were free from systemic disease and were 10 males and 5 females whose ages ranged from 26 to 78 years with a mean of 52.6 ± 15.2 years. None of the control patients had vitreous hemorrhage (Table 1). Vitreous samples were collected undiluted by manual suction into a syringe through the aspiration line of vitrectomy, before opening the infusion line. The samples were centrifuged (5000 rpm for 10 min, 4°C), and the supernatants were aliquoted and frozen at −80°C until assay. The study was conducted according to the tenets of the Declaration of Helsinki, and informed consent was obtained from all patients. The study was approved by the Research Centre, College of Medicine, King Saud University.

### 2.2. Enzyme-Linked Immunosorbent Assay Kits

 Enzyme-linked immunosorbent assay (ELISA) kits for human VEGF (Quantikine Human Vascular Endothelial Growth Factor, Cat number: SVE00), human sVEGFR-2 (Quantikine Human Vascular Endothelial Growth Factor Receptor 2, Cat number: SVR200), human SCF (Quantikine Human Stem Cell Factor/c-kit ligand, Cat number: DCK00), human s-kit (Quantikine Human Stem Cell Factor soluble receptor, SCF sR, Cat number: DSCR00), and eNOS (Quantikine Human Endothelial Nitric Oxide Synthase, Cat number: DEN00) were purchased from R and D Systems, Minneapolis, MN. Whereas, PGE_2_ (Quantikine Human Prostaglandin E2, Cat number: 514010) was purchased from Cayman Chemical Company, Ann Arbor, NI. The detection limit of each ELISA kit for VEGF, sVEGFR-2, SCF, s-kit, eNOS, and PGE_2_ is 9.0, 4.6, 9.0, 65, 25, and 15 picograms/mL (pg/mL), respectively. The ELISA plate readings were done using FLUOstar Omega-Miroplate reader from BMG Labtech, Offenburg, Germany. 

### 2.3. Measurement of Human VEGF, sVEGFR-2, SCF, s-Kit, and eNOS

The quantifications of the level of VEGF, sVEGFR-2, SCF, s-kit, and eNOS in the vitreous fluid were determined using specific ELISA kits according to the manufacturer's instruction (R and D Systems). 

Vitreous samples were diluted 2-fold, 3-fold, and 2-fold for VEGF, sVEGFR-2 and SCF measurements, respectively. 100 *μ*L of diluted sample was added to each well of the ELISA plate for the analysis. For the measurement of s-kit and eNOS, vitreous was not diluted, and 100 *μ*L of undiluted sample was used in the ELISA assay. Optical density was read at 450 nm in microplate reader. Each assay was performed in duplicate. Using the 4-parameter fit logistic (4-PL) curve equation, the actual concentration for each sample was calculated. For the vitreous samples that have been diluted, the correction read from the standard curve obtained using 4-PL was multiplied by the dilution factors to get the actual reading for each sample.

### 2.4. Measurement of Human Prostaglandin E_2_

The quantification of the level of PGE_2_ in the vitreous fluid was determined using a specific ELISA kit according to the manufacturer's instruction (Cayman Chemical Company). 50 *μ*L of 2-fold diluted vitreous fluid was added to each ELISA well followed by the addition of PGE_2_ AChE tracer (acetylcholinesterase) and PGE_2_ monoclonal antibodies. The antibody-PGE_2_ complex binds to goat polyclonal anti-mouse IgG that has been previously attached to the well. The plate was washed after incubation for 18 hours at 4°C to remove any unbound reagents, and then Ellman's Reagent (which contains the substrate to AChE) is added to the well. The product of this enzymatic reaction gives yellow color, and the corresponding optical density was measured at 405 nm using microplate reader. The intensity of this color is proportional to the amount of PGE_2_ tracer bound to the well, which in turn is inversely proportional to the amount of free PGE_2_ present in the well. The %B/B0 (%Bound/Maximum Bound) is calculated which is ratio of the absorbance of a particular sample or standard well to that of the maximum binding (B0) well. Using the 4-parameter fit logistic (4-PL) curve equation, the actual concentration for each sample was calculated using standard curve that was plotted using %B/B0 values versus concentration of a series of wells containing series of known amounts of PGE_2_. As the vitreous fluid has been 2-fold diluted, the correction read from the standard curve obtained using 4-PL was multiplied by the dilution factors to get the actual reading for each sample.

### 2.5. Statistical Analysis

The Mann-Whitney test was used to compare means from two independent groups. Pearson correlation coefficients were computed to investigate correlations between variables. One-way ANOVA and post-ANOVA pairwise comparisons of means were conducted using the Kruskal-Wallis test. For three groups, the critical *Z* value for post-ANOVA pairwise mean comparisons was *Z* = 2.39 at a 5% level of significance. A *P* value less than 0.05 indicated statistical significance. SPSS version 12.0 and program 3S from the BMDP 2007 Statistical Package were used for the statistical analyses.

## 3. Results

### 3.1. Levels of Angiogenic and Vasculogenic Factors in Vitreous Samples

VEGF was detected in all vitreous samples from patients with PDR and in 8 (53.0%) samples from nondiabetic control patients (Table 1). When all patients were considered, mean VEGF level in vitreous samples from PDR patients (711.6 ± 1271.3 pg/mL) was significantly higher than that in nondiabetic control patients (31.6 ± 37.9 pg/mL) (*P* < 0.001; Mann-Whitney test). 

sVEGFR-2 was detected in all vitreous samples from patients with PDR and nondiabetic control patients (Table 1). The detected sVEGFR-2 levels in PDR patients (1497.0 ± 1590.5 pg/mL) were significantly higher than that in nondiabetic control patients (678.0 ± 471.5 pg/mL) (*P* = 0.002; Mann-Whitney test). 

SCF was detected in all vitreous samples from patients with PDR and in 5 (33.0%) samples from nondiabetic control patients (Table 1). SCF mean level in vitreous samples from PDR patients (176.6 ± 490.1 pg/mL) was significantly higher than that in nondiabetic control patients (21.6 ± 38.5 pg/mL) (*P* < 0.001; Mann-Whitney test).

s-kit was detected in all vitreous samples from patients with PDR and in 8 (53.0%) samples from nondiabetic control patients (Table 1). s-kit mean level in vitreous samples from PDR patients (493.8 ± 1183.1 pg/mL) was significantly higher than that in nondiabetic control patients (87.5 ± 91.5 pg/mL) (*P* < 0.001; Mann-Whitney test).

PGE_2_ was detected in all vitreous samples from patients with PDR and nondiabetic control patients (Table 1). The detected PGE_2_ levels in PDR patients (28.7 ± 10.6 pg/mL) were significantly lower than that in nondiabetic control patients (58.0 ± 18.8 pg/mL) (*P* < 0.001; Mann-Whitney test). eNOS was not detected in vitreous samples from patients with PDR and nondiabetic control patients.

### 3.2. Relationship between Angiogenic and Vasculogenic Factors and Activity of PDR

 Comparison of mean levels of angiogenic and vasculogenic factors among active PDR patients, inactive PDR patients, and nondiabetic control patients was conducted using the Kruskal-Wallis test, and the results are shown in Table 2. Mean levels differed significantly between the 3 groups for VEGF (*P* < 0.001), sVEGFR-2 (*P* = 0.007), SCF (*P* = 0.001), s-kit (*P* < 0.001), and PGE_2_ (*P* < 0.001). Post-ANOVA pairwise comparisons of means indicated that mean VEGF level was significantly higher in patients with active PDR than in patients with inactive PDR (*Z* = 3.67) and nondiabetic control patients (*Z* = 5.25). For sVEGFR-2, mean level for patients with active PDR was significantly higher than that in nondiabetic control patients (*Z* = 3.11). For SCF, the mean levels for patients with active PDR and patients with inactive PDR were significantly higher than that in nondiabetic control patients (*Z* = 3.42; *Z* = 3.02, resp.). For s-kit, the mean level for patients with active PDR was significantly higher than that in nondiabetic control patients (*Z* = 4.0). For PGE_2_, the mean levels for patients with active PDR and patients with inactive PDR were significantly lower than that in nondiabetic control patients (*Z* = 3.73; *Z* = 2.73, resp.).

### 3.3. Relationship between Angiogenic and Vasculogenic Factors and Vitreous Hemorrhage

 When patients with PDR were divided into those with or without hemorrhage, the mean levels of angiogenic and vasculogenic factors differed significantly between PDR patients with hemorrhage, PDR patients without hemorrhage, and nondiabetic control patients for VEGF (*P* < 0.001), sVEGFR-2 (*P* = 0.003), SCF (*P* = 0.001), s-kit (*P* = 0.001), and PGE_2_ (*P* < 0.001) (Table 3). Post-ANOVA pairwise comparisons of means highlighted that for VEGF, the mean levels for PDR patients with or without hemorrhage were significantly higher than those for nondiabetic control patients (*Z* = 4.75; *Z* = 2.52, resp.), and the mean level for PDR patients with hemorrhage was significantly higher than that for PDR patients without hemorrhage (*Z* = 2.40). For sVEGFR-2, the mean levels for PDR patients with or without hemorrhage were significantly higher than those for nondiabetic control patients (*Z* = 3.37; *Z* = 2.45). For SCF, the mean levels for PDR patients with or without hemorrhage were significantly higher than those for nondiabetic control patients (*Z* = 3.27; *Z* = 3.21, resp.). For s-kit, the mean levels for PDR patients with or without hemorrhage were significantly higher than those for nondiabetic control patients (*Z* = 3.5; *Z* = 2.88, resp.). For PGE_2_, the mean level was significantly lower for PDR patients with hemorrhage than that for nondiabetic control patients (*Z* = 4.23).

There were no statistically significant relationships between vitreous levels of angiogenic and vasculogenic factors and systemic disease variables (Table 4).

### 3.4. Correlations

 When all patients with diabetes were considered, there were significant correlations between vitreous fluid levels of sVEGFR-2 versus SCF (*r* = 0.950, *P* < 0.001), sVEGFR-2 versus s-kit (*r* = 0.941, *P* < 0.001), and SCF versus s-kit (*r* = 0.970, *P* < 0.001) (Table 5).

## 4. Discussion

 The present study is to our knowledge the first to assess the levels of sVEGFR-2, SCF, and s-kit in the vitreous fluid from patients with PDR. Because activation of VEGFR-2 plays an important role in tumor angiogenesis, clinical interest in monitoring plasma sVEGFR-2 levels in cancer patients has focused on its potential use as a surrogate biomarker for disease progression as well as assessing efficacy/activity of antiangiogenic drugs particularly those that target VEGF or VEGFR-2 [9, 27]. In vitro studies showed that VEGFR-2 downregulation from the cell surface leads to reduced sVEGFR-2 levels in the conditioned media from endothelial cells, and that sVEGFR-2 is derived mainly from shedding from endothelial cell surface. These findings imply that expression levels of VEGFR-2 and its soluble form are linked [9]. Therefore, increased levels of sVEGFR-2 in the vitreous from patients with PDR, particularly in patients with active PDR, may reflect increased cellular VEGFR-2. A VEGF-dependent increase in the shedding of sVEGFR-2 by endothelial cells was demonstrated [28, 29]. In the present study, we detected higher levels of VEGF in patients with active PDR compared with patients with inactive PDR and nondiabetic patients. Collectively, these findings are consistent with our previous immunohistochemical studies in which we demonstrated the presence of VEGFR-2^+^ CD34^+^ cells and VEGF^+^ cells in the vascular endothelium of blood vessels and in the stroma in PDR fibrovascular epiretinal membranes. Furthermore, the numbers of blood vessels and stromal cells expressing VEGFR-2 and VEGF in membranes from patients with active PDR were significantly higher than that in membranes from patients with quiescent PDR [3, 30].

 In a previous immunohistochemical study, we showed that bone marrow-derived c-kit^+^ cells coexpressing the chemokine stromal cell-derived factor-1 receptor CXCR4 and eNOS contribute to new vessel formation in PDR fibrovascular epiretinal membranes. In addition, SCF was expressed by vascular endothelial cells and stromal cells. Furthermore, the expression of SCF and c-kit in membranes from patients with active neovascularization was significantly higher than that in membranes from patients with quiescent PDR [4]. We have extended those observations by showing that both SCF and s-kit levels were significantly elevated in vitreous from patients with PDR and were further increased in patients with active PDR. Taken together, these findings suggest a role for the SCF/c-kit signaling in the progression of PDR.

 Several reports demonstrated that SCF/c-kit signaling axis has been implicated in the regulation of angiogenesis. SCF/c-kit signaling promoted the survival, migration, differentiation, and capillary tube formation of endothelial cells [16, 17] and induced a potent angiogenic response in vivo [17]. In addition, SCF/c-kit signaling played an important role in ischemia-induced neovascularization [10, 13, 15, 18]. SCF has also been implicated in the mobilization of bone marrow-derived endothelial progenitor cells required for neovascularization [12, 15]. Interestingly, several studies showed that SCF/c-kit signaling upregulates the transcription factor hypoxia-inducible factor-1*α* (HIF-1*α*). SCF-induced HIF-1*α* was transcriptionally active and transcribed HIF-1*α* target genes, such as VEGF [31, 32]. On the other hand, Han et al. [33] demonstrated that hypoxia upregulates SCF gene expression through HIF-1*α*. These findings suggest a reciprocal effect between SCF and HIF-1*α*, thus forming a positive feedback in several cell lines coexpressing SCF and c-kit. In a previous report we demonstrated the presence of HIF-1*α* immunoreactivity in PDR epiretinal membranes [34]. Treatment of small cell lung cancer cell line with imatinib, a specific inhibitor of the protein tyrosine kinases c-kit, and platelet-derived growth factor receptor resulted in inhibition of c-kit-induced HIF-1*α* and VEGF expression [31]. Imatinib has been shown to have clinical activity as an anticancer agent [35]. These findings suggest that inhibition of SCF/c-kit signaling could have clinically relevant antiangiogenic effects in PDR. 

 Several studies demonstrated that circulating levels of s-kit correlate with the clinical course of tumors and that the concentration of s-kit may be a useful clinical biomarker of disease severity in patients with tumors [36, 37]. The serum levels of s-kit increase when the population of cells that release c-kit is pathologically expanded such as in acute myelogenous leukemia and mastocytosis [38, 39]. Because s-kit is thought to be generated by the proteolytic cleavage of the membrane-bound receptor [19], it is possible that the increased levels in the vitreous from patients with PDR in part reflect increased c-kit^+^ cell numbers in PDR fibrovascular epiretinal membranes [4]. In addition, Nakamura et al. [40] demonstrated that exogenous s-kit induces mobilization of hematopoietic stem cells from bone marrow into peripheral blood. This finding is consistent with the observations of other investigators who demonstrated that the levels of s-kit in the serum showed a positive correlation with the numbers of peripheral blood stem cells [41]. The present study showed positive correlations between vitreous levels of SCF, s-kit, and sVEGFR-2. Similarly, c-kit and VEGFR-2 amplifications were strongly associated in glioblastoma multiforme, suggesting coamplification [42]. On the other hand, Turner et al. [43] demonstrated that s-kit retains high-affinity SCF binding activity suggesting that s-kit may bind SCF and function as a receptor antagonist.

 eNOS is required for neovascularization in ischemic tissue [22, 23]. In a mice model of hind-limb ischemia, impaired neovascularization in mice lacking eNOS is related to a defect in progenitor cell mobilization [22]. eNOS is involved in migration of EPCs [21] and is crucial and specific factor for firm c-kit^+^ cell adhesion in the vascular endothelium [20]. In cell culture models, eNOS plays an essential role in endothelial cell proliferation and is a central mediator of several endothelium growth factors, such as VEGF and PGE_2_ [44]. In a previous immunohistochemical study, we showed immunoreactivity for eNOS in vascular endothelial cells and stromal cells in PDR fibrovascular epiretinal membranes [4]. In the present study, however, eNOS levels were below the detection limit. This discrepancy might be explained by the primary localization of eNOS on the Golgi apparatus and plasma membrane caveolae in endothelial cells [45] and therefore might not release into the vitreous fluid. The crucial role of PGE_2_ in EPCs homeostasis following tissue ischemia has been demonstrated. In vivo blockade of PGE_2_ production by selective cyclooxygenase-2 inhibition virtually abrogated ischemia-induced EPCs mobilization. In addition, EPCs are a rich source of PGE_2_, and PGE_2_ stimulates the number and function of EPCs [24]. Furthermore, PGE_2_ directly stimulates angiogenesis, and this stimulatory effect is not dependent on VEGF signaling. PGE_2_ stimulation of endothelial cells enhances VEGF expression. In turn, VEGF stimulates PGE_2_ expression in endothelial cells [25]. However, in the present study, PGE_2_ levels in PDR patients were significantly lower than that in nondiabetic patients. These findings are consistent with the observations of other investigators [46].

The vitreous fluid, collected from patients with PDR during pars plana vitrectomy, is an ideal material for analysis of local, intraocular concentrations of selected proteins which take part of this pathology. However, when measuring these factors in the vitreous, some considerations should be kept in mind. Vitreous hemorrhage, associated with active neovascularization or traction on the retina induced by involuted fibrovascular proliferation during posterior vitreous detachment, can provide an influx of serum proteins into vitreous fluid. In a previous study, we demonstrated that there was no correlation between hemoglobin levels, as a measure of the amount of erupted blood, and total protein levels in vitreous fluid from patients with PDR [47]. In addition, we showed that chemokine levels in vitreous samples were significantly higher than that in serum samples and that there was no correlation between vitreous fluid and serum chemokine levels in patients with PDR [48]. Our previous immunohistochemical studies demonstrated the expression of c-kit, SCF, VEGF, and VEGFR-2 by vascular endothelial cells and stromal cells in PDR fibrovascular epiretinal membranes [3, 4, 30, 34]. In our laboratory, we also recently demonstrated upregulated expression of VEGF, VEGFR-2, and SCF in the retinas from diabetic mice (Mohammed et al., unpublished data). In addition, our subgroup analysis demonstrated that the levels of VEGF, sVEGFR-2, SCF, and s-kit were also increased in vitreous fluid from PDR patients without hemorrhage. These findings suggest that local cellular production is the relevant source of these factors within the ocular microenvironment and that systemic inflow mechanism is rather improbable.

 In conclusion, our findings suggest that the upregulation of VEGF, sVEGFR-2, SCF, and s-kit in the vitreous fluid from patients with PDR reflects angiogenesis and vasculogenesis in PDR. These results have implications for understanding the pathogenetic mechanisms that underlie the neovascularization that develops as a complication of this disorder.

## Figures and Tables

**Table 1:** Clinical characteristics and levels of angiogenic and vasculogenic factors in 15 control patients.

Age (yr)/sex	Duration of symptoms (days)	Status of lens	Extent of detachment (quadrants)	VEGF (pg/mL) (detection limit: 9.0 pg/mL)	sVEGFR-2 (pg·mL) (detection limit: 4.6 pg/mL)	SCF (pg/mL) (detection limit: 9.0 pg/mL)	s-Kit (pg/mL) (detection limit: 65 pg/mL)	PGE_2_ (pg/mL) (detection limit: 25 pg/mL)	eNOS (pg/mL) (detection limit: 15 pg/mL)
78/M	4	Pseudophakic	2	19.3	837.4	ND	87	68.2	ND
60/F	3	Pseudophakic	2	38.8	161.2	ND	ND	78.8	ND
47/M	6	Pseudophakic	4	ND	1126.6	128.4	247	38	ND
48/F	5	Phakic	2	ND	1730.7	ND	ND	42.8	ND
59/M	3	Phakic	2	ND	1016.2	22.2	163	80.2	ND
38/F	14	Phakic	2	33.1	93	ND	ND	28.1	ND
40/M	5	Phakic	3	ND	915.3	59.1	135	20.7	ND
27/F	7	Phakic	2	ND	656.2	ND	224.9	70.8	ND
65/F	10	Pseudophakic	4	27.7	783	ND	173	63.9	ND
55/M	2	Phakic	2	26.4	304.4	ND	ND	45	ND
40/F	4	Aphakic	2	ND	912.5	ND	ND	61.3	ND
70/M	2	Pseudophakic	2	16.5	934.4	37.6	58	67.3	ND
74/M	3	Pseudophakic	1	21	664.4	ND	137	63.2	ND
63/M	2	Pseudophakic	2	30.8	140.7	ND	ND	80.3	ND
50/M	5	Pseudophakic	3	ND	731.2	76.7	ND	61.7	ND

VEGF: vascular endothelial growth factor; sVEGFR-2: soluble vascular endothelial growth factor receptor-2; SCF: stem cell factor; s-kit: soluble c-kit; PGE_2_: prostaglandin E_2_; eNOS: endothelial nitric oxide synthase; ND: not detected.

**Table 2:** Comparisons of mean angiogenic and vasculogenic factor levels in proliferative diabetic retinopathy (PDR) patients with or without active neovascularization.

Disease group	VEGF (pg/mL)	sVEGFR-2 (pg/mL)	SCF (pg/mL)	s-kit (pg/mL)	PGE_2_ (pg/mL)
Active PDR	1099.9 ± 1535.6	1692.5 ± 1873.9	233.7 ± 626.8	697.4 ± 1528.1	28.2 ± 11.1
Inactive PDR	150.7 ± 237.7	1050.1 ± 403.2	88.6 ± 66.2	205.3 ± 106.4	30.0 ± 10.1
Controls	31.6 ± 37.9	678.0 ± 471.5	21.6 ± 38.5	87.5 ± 91.5	58.0 ± 18.8
ANOVA *P* value	<0.001*	0.007*	0.001*	<0.001*	<0.001*

*Statistically significant at 5% level.

VEGF: vascular endothelial growth factor; sVEGFR-2: soluble vascular endothelial growth factor receptor-2; SCF: stem cell factor; s-kit: soluble c-kit; PGE_2_: prostaglandin E_2_.

**Table 3:** Comparisons of mean angiogenic and vasculogenic factor levels in proliferative diabetic retinopathy (PDR) patients with or without hemorrhage.

Disease group	VEGF (pg/mL)	sVEGFR-2 (pg/mL)	SCF (pg/mL)	s-kit (pg/mL)	PGF_2_ (pg/mL)
PDR with hemorrhage	994.1 ± 1357.4	1567.9 ± 2118.8	253.1 ± 660.2	682.7 ± 1643.0	24.6 ± 9.3
PDR without hemorrhage	453.6 ± 1156.7	1404.7 ± 425.8	84.7 ± 56.4	291.4 ± 149.3	34.1 ± 10.3
Controls	31.6 ± 37.9	678.0 ± 471.5	21.6 ± 38.5	87.5 ± 91.5	58.0 ± 18.8
ANOVA *P* value	<0.001*	0.003*	0.001*	0.001*	<0.001*

*Statistically significant at 5% level.

VEGF: vascular endothelial growth factor; sVEGFR-2: soluble vascular endothelial growth factor receptor-2; SCF: stem cell factor; s-kit: soluble c-kit; PGE_2_: prostaglandin E_2_.

**Table 4:** Relationship between angiogenic and vasculogenic factors and systemic disease variables.

Variable	VEGF (pg/mL)	sVEGFR-2 (pg/mL)	SCF (pg/mL)	s-kit (pg/mL)	PGE_2_ (pg/mL)
Type of diabetes					
Insulin-dependent	1125.4 ± 1819.4	1594.0 ± 1950.7	209.9 ± 614.4	601.3 ± 1502.7	29.6 ± 10.6
Noninsulin-dependent	472.9 ± 507.9	1338.2 ± 569.3	116.5 ± 75.5	320.3 ± 182.6	28.8 ± 11.1
*P* value	0.611	0.698	0.118	0.578	0.972
Fasting blood glucose					
Controlled	1296.1 ± 1115.8	2133.0 ± 2601.7	313.4 ± 809.2	867.0 ± 2014.4	31.0 ± 11.0
Uncontrolled	912.9 ± 1815.2	1158.1 ± 504.8	906 ± 74.2	297.9 ± 177.0	28.4 ± 10.5
*P* value	0.081	0.375	0.846	0.649	0.733
Hypertension					
Yes	1117.3 ± 1757.7	1639.4 ± 1873.0	209.4 ± 573.0	548.0 ± 1358.0	29.4 ± 10.6
No	672.6 ± 1063.8	1008.8 ± 535.1	59.7 ± 28.0	330.6 ± 196.2	29.2 ± 12.5
*P*-value	0.374	0.409	0.422	0.716	0.804

VEGF: vascular endothelial growth factor; sVEGFR-2: soluble vascular endothelial growth factor receptor-2; SCF: stem cell factor; s-kit: soluble c-kit; PGE_2_: prostaglandin E_2_.

**Table 5:** Pearson correlation coefficients.

	PGE_2_	VEGF	sVEGFR-2	SCF
VEGF				
*r* =	0.054			
*P* =	0.849			
sVEGFR-2				
*r* =	−0.060	−0.151		
*P* =	0.785	0.590		
SCF				
*r* =	−0.085	−0.002	0.950	
*P* =	0.699	0.991	<0.001*	
s-kit				
*r* =	0.001	−0.021	0.941	0.970
*P* =	0.998	0.926	<0.001*	<0.001*

*Statistically significant at 5% level.

Where the row and column meet, is the correlation coefficient and the *P* value for the two variables.

VEGF: vascular endothelial growth factor; sVEGFR-2: soluble vascular endothelial growth factor receptor-2; SCF: stem cell factor; s-kit: soluble c-kit; PGE_2_: prostaglandin E_2_.
